# Mobility in Complex Environments: Investigating the Differences in Aging Trajectories

**DOI:** 10.1093/geroni/igaf122.2114

**Published:** 2025-12-31

**Authors:** Sudeshna Chatterjee, David Clark, Patricia Shewokis, Lynnette Montgomery, Jennifer Plumb, Diana Robins, Hasan Ayaz

**Affiliations:** Drexel University, Philadelphia, Pennsylvania, United States; University of Florida, Gainesville, Florida, United States; Drexel University, Philadelphia, Pennsylvania, United States; Drexel University, Philadelphia, Pennsylvania, United States; Drexel University, Philadelphia, Pennsylvania, United States; Drexel University, Philadelphia, Pennsylvania, United States; Drexel University, Philadelphia, Pennsylvania, United States

## Abstract

Age-related decline in executive function, balance, and movement control are associated with fall risk, activity avoidance, and lower engagement in physical activity which may restrict functional independence and participation in life roles. The deficits in control mechanisms are particularly amplified in complex environments at home and in the community, such as uneven surfaces, poor ambient conditions, and/or distractions. Nearly 35% of older adults report falling in an outdoor environment, and 59% of the falls occur due to trips and slips. Using mobile brain imaging with functional near-infrared spectroscopy, we have reported that age, executive function, and their interaction are associated with the magnitude of prefrontal brain recruitment during obstacle negotiation (N = 50, R²=0.34, p = 0.01). Specifically, lower executive function in adults aged <75 years was associated with greater prefrontal recruitment during obstacle negotiation when compared to peers with higher executive function (p = 0.03). Interestingly, older adults aged ≥75 years exhibited a ceiling of prefrontal recruitment during obstacle negotiation, regardless of their executive function (p = 0.87). It is currently not clear how aging with neurodevelopmental conditions such as autism impacts mobility in complex environments. Since adults with autism are twice as susceptible to injurious falls as their peers and have a higher risk of developing conditions such as Parkinson’s disease and dementia, we are investigating the differences in executive function, balance, and movement control in adults with autism aged 18 to 64 years. Our findings will provide novel insights into mobility function in this understudied population and guide future lifestyle interventions.

